# Metatranscriptomics by *In Situ* RNA Stabilization Directly and Comprehensively Revealed Episymbiotic Microbial Communities of Deep-Sea Squat Lobsters

**DOI:** 10.1128/mSystems.00551-20

**Published:** 2020-10-06

**Authors:** Kaori Motoki, Tomo-o Watsuji, Yoshihiro Takaki, Ken Takai, Wataru Iwasaki

**Affiliations:** a Department of Biological Sciences, Graduate School of Science, The University of Tokyo, Bunkyo-ku, Tokyo, Japan; b Institute for Extra-cutting-edge Science and Technology Avant-garde Research (X-star), Super-cutting-edge Grand and Advanced Research (SUGAR) Program, Japan Agency for Marine-Earth Science and Technology (JAMSTEC), Yokosuka, Kanagawa, Japan; c Department of Food and Nutrition, Higashi Chikushi Junior College, Kitakyushu, Fukuoka, Japan; d Department of Computational Biology and Medical Sciences, Graduate School of Frontier Sciences, The University of Tokyo, Kashiwa, Chiba, Japan; e Atmosphere and Ocean Research Institute, The University of Tokyo, Kashiwa, Chiba, Japan; f Institute for Quantitative Biosciences, The University of Tokyo, Bunkyo-ku, Tokyo, Japan; g Collaborative Research Institute for Innovative Microbiology, The University of Tokyo, Bunkyo-ku, Tokyo, Japan; University of Technology Sydney

**Keywords:** chemosynthesis, deep sea, *in situ* analysis, metatranscriptome, symbiosis

## Abstract

Shinkaia crosnieri is an invertebrate that inhabits an area around deep-sea hydrothermal vents in the Okinawa Trough in Japan by harboring episymbiotic microbes as the primary nutrition. To reveal physiology and phylogenetic composition of the active episymbiotic populations, metatranscriptomics is expected to be a powerful approach. However, this has been hindered by substantial perturbation (e.g., RNA degradation) during time-consuming retrieval from the deep sea. Here, we conducted direct metatranscriptomic analysis of S. crosnieri episymbionts by applying *in situ* RNA stabilization equipment. As expected, we obtained RNA expression profiles that were substantially different from those obtained by conventional metatranscriptomics (i.e., stabilization after retrieval). The episymbiotic community members were dominated by three orders, namely, *Thiotrichales*, *Methylococcales*, and *Campylobacterales*, and the *Campylobacterales* members were mostly dominated by the *Sulfurovum* genus. At a finer phylogenetic scale, the episymbiotic communities on different host individuals shared many species, indicating that the episymbionts on each host individual are not descendants of a few founder cells but are horizontally exchanged. Furthermore, our analysis revealed the key metabolisms of the community: two carbon fixation pathways, a formaldehyde assimilation pathway, and utilization of five electron donors (sulfide, thiosulfate, sulfur, methane, and ammonia) and two electron accepters (oxygen and nitrate/nitrite). Importantly, it was suggested that *Thiotrichales* episymbionts can utilize intercellular sulfur globules even when sulfur compounds are not usable, possibly also in a detached and free-living state.

**IMPORTANCE** Deep-sea hydrothermal vent ecosystems remain mysterious. To depict in detail the enigmatic life of chemosynthetic microbes, which are key primary producers in these ecosystems, metatranscriptomic analysis is expected to be a promising approach. However, this has been hindered by substantial perturbation (e.g., RNA degradation) during time-consuming retrieval from the deep sea. In this study, we conducted direct metatranscriptome analysis of microbial episymbionts of deep-sea squat lobsters (*Shinkaia crosnieri*) by applying *in situ* RNA stabilization equipment. Compared to conventional metatranscriptomics (i.e., RNA stabilization after retrieval), our method provided substantially different RNA expression profiles. Moreover, we discovered that *S. crosnieri* and its episymbiotic microbes constitute complex and resilient ecosystems, where closely related but various episymbionts are stably maintained by horizontal exchange and partly by their sulfur storage ability for survival even when sulfur compounds are not usable, likely also in a detached and free-living state.

## INTRODUCTION

In environments without sunlight, ecosystems and geochemical cycles are often powered by chemosynthetic microbes that utilize reduced chemical compounds as energy sources (1–3). As a notable example, deep-sea hydrothermal vents can harbor an abundance of animals that actively proliferate by obtaining nutrient sources from their symbiotic chemosynthetic microbes (4). Beginning with the discovery of tubeworm Riftia pachyptila associated with microbes oxidizing reduced sulfur compounds in 1981 (5–7), symbioses with chemosynthetic microbes have been found for various invertebrate phyla, including Mollusca, Annelida, and Arthropoda, and their microbial symbionts are either inside (endosymbionts) or outside (episymbionts) the host bodies (8, 9). To obtain comprehensive and detailed knowledge on the taxonomic composition and metabolic activities of these microbes, a metatranscriptomic approach is expected to be promising (10); however, fundamental obstacles exist in the application of this approach to deep-sea environments (11).

Shinkaia crosnieri is a deep-sea invertebrate that predominantly (up to 465 individuals/m^2^) inhabits an area around hydrothermal vents in the Okinawa Trough in Japan (12). S. crosnieri utilizes episymbiotic microbes on the surface of its ventral setae as its primary nutrient source, and its symbiotic system is the most clearly characterized one among deep-sea episymbioses (13, 14). Those episymbionts were revealed to contain methane-utilizing and sulfur (thiosulfate and sulfide)-oxidizing chemolithotrophic bacteria (15, 16). Phylogenetically, 16S rRNA gene clone analysis reproducibly identified species belonging to the genus *Sulfurovum* in *Epsilonproteobacteria* (recently proposed to be renamed as Campylobacterota [17–19]) and the orders *Thiotrichales* and *Methylococcales* in *Gammaproteobacteria* as major episymbionts (20, 21). Of these episymbionts, fluorescence *in situ* hybridization coupled to nanoscale secondary ion mass spectrometry (FISH-NanoSIMS) revealed that the *Sulfurovum* episymbionts oxidize reduced sulfur compounds and fix inorganic carbon (15). The *Thiotrichales* and *Methylococcales* episymbionts were inferred to be thioautotrophs and methanotrophs, respectively, based on the physiology of related species and reverse transcription-PCR (RT-PCR)/phylogenetic analyses of *pmoA*, a key gene for methane oxidation (16). However, experimental investigation of the *S. crosnieri* episymbionts in laboratories is impeded because of difficulties in culturing these microbes and rearing the hosts by accurately simulating hydrothermal vent environments. Rearing *S. crosnieri* in a tank supplied with sulfide or methane was successfully conducted, but this process substantially changed the episymbiotic microbial community composition (22, 23).

Given these difficulties in laboratory experiments, metatranscriptomics by *in situ* RNA stabilization is expected to be promising for direct investigation of these episymbionts as mentioned above. However, in addition to the apparent difficulty of sampling from the deep sea itself, the time and environmental changes required by sampling processes constitute fundamental obstacles, although most of the metatranscriptomic studies of symbiotic and free-living deep-sea microbes stabilized RNA after onboard recovery (11, 24). During deep-sea sampling and onboard recovery, which usually takes more than an hour, the composition and activity of microbial communities can change considerably, while RNAs can be degraded (25), which means that true RNA profiles in the deep sea can be missed if experimental procedures are conducted after retrieval (26, 27). To solve these problems, *in situ* RNA stabilization (i.e., stabilization before retrieval by remote control) instead of onboard RNA stabilization (i.e., stabilization after retrieval) is desirable (16, 28–32); however, these two approaches have been neither systematically compared nor applied to metatranscriptomic analyses of deep-sea symbiotic microbial communities.

In this study, we applied *in situ* and onboard RNA stabilization methods to 16S rRNA and metatranscriptomic analyses of the *S. crosnieri* episymbiotic microbial communities to (i) delineate their taxonomic composition by increasing sensitivity and eliminating biases in PCR cloning, (ii) systematically estimate the effects of the sampling processes from deep-sea hydrothermal vents that are characterized by unusual chemical and physical conditions, and (iii) obtain comprehensive knowledge on their physiology.

## RESULTS AND DISCUSSION

### Total RNA (rRNA) analysis of *S. crosnieri* episymbiotic microbial communities.

*S. crosnieri* sampling was conducted at a 982-m-deep hydrothermal vent site in the Iheya North field in the Okinawa Trough in Japan (see Fig. S1 in the supplemental material) in 2015, and episymbiotic microbial community samples subjected to RNA stabilization either *in situ* or onboard were obtained from the host setae. Total RNA sequencing with Ion PGM System of six *in situ* and seven onboard RNA-stabilized samples from different host individuals yielded 5,430,057 and 4,847,064 reads, respectively (Table 1). After filtering short and low-quality reads, 619,694 and 695,480 rRNA reads, respectively, were used for taxonomic analyses.

**TABLE 1:** Sequencing of total RNA

Stabilization method	Sample ID	Specimen ID	No. of raw reads	No. of quality- controlled reads	Mean read length (bp)	No. of mapped reads
*In situ*	*In situ* total RNA 1	6	431,185	36,143	130.5	7,714
	*In situ* total RNA 2	8	1,276,721	143,663	134.4	27,636
	*In situ* total RNA 3	9	1,109,657	133,618	142.4	25,482
	*In situ* total RNA 4	10	695,387	129,258	158.7	38,269
	*In situ* total RNA 5	11	623,241	83,526	142.8	27,014
	*In situ* total RNA 6	12	1,293,866	93,486	129.4	25,424

Onboard	Onboard total RNA 1	1	426,120	79,465	155.5	16,378
	Onboard total RNA 2	2	759,925	66,155	128.8	15,304
	Onboard total RNA 3	7	567,413	97,520	153.4	38,240
	Onboard total RNA 4	8	607,935	100,741	152.2	23,636
	Onboard total RNA 5	9	1,041,853	192,140	152.2	58,984
	Onboard total RNA 6	10	776,235	58,905	123.2	16,047
	Onboard total RNA 7	11	667,583	100,554	146.3	16,894

10.1128/mSystems.00551-20.1 FIG S1 Location of the Iheya North hydrothermal field, Okinawa Trough, in Japan. The figure is based on a map made with Natural Earth (naturalearthdata.com). Download FIG S1, EPS file, 1.1 MB. Copyright © 2020 Motoki et al. 2020 Motoki et al. This content is distributed under the terms of the Creative Commons Attribution 4.0 International license.

**FIG 1:**
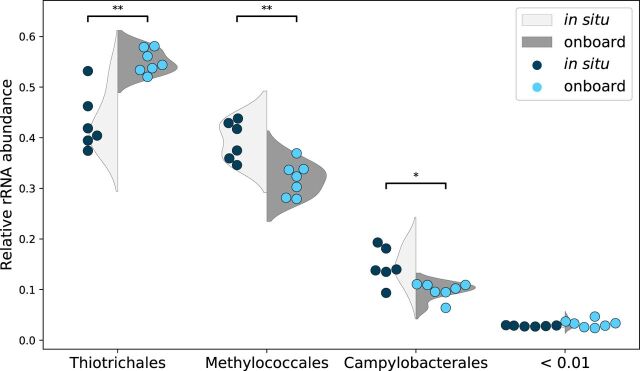
Relative rRNA abundances at the order level. Three major orders for which the relative abundances of rRNA expression were >0.01 in all samples are shown. Each symbol shows the value for a sample (six *in situ* and seven onboard), and violin plots are presented for reference. Mann-Whitney *U* tests were performed (**, *P* < 0.01; *, *P* < 0.05).

The relative abundances of the rRNA reads confirmed that the orders *Thiotrichales*, *Methylococcales*, and *Campylobacterales* were the major community members. These three major orders showed relative abundances of >5% in every sample and accounted for 95.3% to 97.6% in total (Fig. 1). At the genus level, *Sulfurovum* was particularly dominant and accounted for more than 99% of the *Campylobacterales* rRNA reads in all samples (see Table S1 in the supplemental material). Notably, *Thiotrichales*, *Methylococcales*, and *Sulfurovum* sequences were consistently found in a previous study that applied a PCR-based method to *S. crosnieri* episymbiotic microbial communities at the same site in 2007 (20). Therefore, *Thiotrichales*, *Methylococcales*, and *Campylobacterales* (or *Sulfurovum*) seemed to be stable as major members in *S. crosnieri* episymbiotic communities at least at this hydrothermal vent site, irrespective of possible PCR biases.

10.1128/mSystems.00551-20.5 TABLE S1 Summary of rRNA reads assigned to genera within *Campylobacterales* (relative abundance [read numbers]). Download Table S1, DOCX file, 0.01 MB. Copyright © 2020 Motoki et al. 2020 Motoki et al. This content is distributed under the terms of the Creative Commons Attribution 4.0 International license.

**FIG 2:**
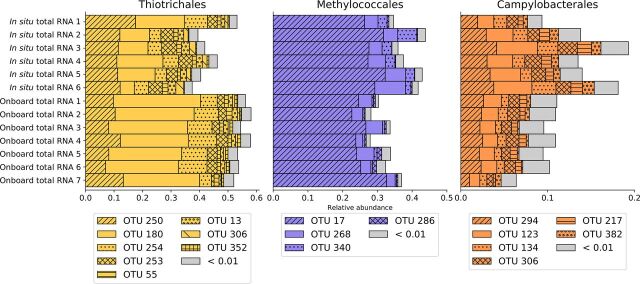
Relative rRNA abundances at the OTU level. OTUs clustered at the 95% identity threshold for which the relative abundances of rRNA expression were >0.01 in all samples are shown.

The *Thiotrichales*, *Methylococcales*, and *Campylobacterales* rRNA sequences contained 199, 132, and 337 SILVA identifiers, which were clustered to 82, 44, and 85 operational taxonomic units (OTUs), respectively, at a 95% similarity threshold (Fig. 2). These numbers suggest that each of the three *S. crosnieri* episymbiont taxa comprised dozens of species instead of a few specifically adapted species. The OTU compositions were highly similar among the different *S. crosnieri* individuals. The *Thiotrichales* and *Methylococcales* episymbionts contained two (OTUs 250 and 180) and one (OTU 17) abundant OTU, respectively. On the other hand, no specific dominant OTUs were observed in the *Campylobacterales* (*Sulfurovum*) episymbionts. The saturation of rarefaction curves for all samples suggested that the entire episymbiotic microbial communities were well sampled (Fig. S3a). Collectively, the large numbers of OTUs observed in each sample and the community composition similarities among the samples indicate that the episymbiotic community of each *S. crosnieri* individual is not composed of clonal descendants of a few “founder” cells that settled down after birth or molting of the host but is more complex than previously thought and maintained by horizontal exchange between host individuals around the hydrothermal vent.

### Episymbiotic microbial community comparisons between the *in situ* and onboard RNA stabilization methods.

While OTU richness and Faith’s phylogenetic diversity were not significantly different between the *in situ* and onboard RNA-stabilized samples (*P* > 0.05, Mann-Whitney *U* test; Fig. S3b and c), the *in situ* RNA-stabilized samples showed significantly larger Shannon diversity (*P* = 0.018, Mann-Whitney *U* test; Fig. S3d). Thus, while the OTU sets of the episymbiotic communities were not different between the sampling methods, skewness in their abundances decreased during the retrieval process from the deep sea. Weighted UniFrac and the principal coordinate analysis (PCoA) revealed that the *in situ* and onboard RNA-stabilized data were significantly different (*P* = 0.002, permutational multivariate analysis of variance [PERMANOVA], 999 permutations in each test, Fig. S4). It may be noted that the composition of the *in situ* total RNA 1 sample was exceptionally close to that of the onboard stabilized samples, but this sample was also characterized by the particularly small number of the mapped reads and OTUs, likely because of problems in sequencing (Table 1). We confirmed that exclusion of *in situ* total RNA 1 sample did not affect the results of the statistical tests of the OTU richness, Faith’s phylogenetic diversity, and Shannon diversity.

Comparison of the relative abundances between the *in situ* and onboard RNA stabilization methods showed relative increases in *Thiotrichales* (4.0% on average) and decreases in *Methylococcales* (11.9% on average) and *Campylobacterales* (6.5% on average) in the onboard stabilization samples, and these shifts were consistently observed regardless of OTUs among different host individuals (Fig. 2). Thus, as expected, the approximately 3-h sampling process without RNA stabilization likely affected the rRNA abundances of the major taxa in a systematic manner. In particular, *Thiotrichales* OTU 180 (corresponding to the genus *Thiothrix*) showed a substantial increase under onboard RNA stabilization (Table S2). Notably, *Thiothrix* was reported to store reduced sulfur compounds as intercellular sulfur granules and utilize them as energy sources by oxidization to sulfate (discussed later) (33–35).

10.1128/mSystems.00551-20.6 TABLE S2 Detected SILVA identifiers and OTUs. Download Table S2, DOCX file, 0.01 MB. Copyright © 2020 Motoki et al. 2020 Motoki et al. This content is distributed under the terms of the Creative Commons Attribution 4.0 International license.

### Metatranscriptomic analysis of *S. crosnieri* episymbiotic microbial communities.

rRNA-depleted RNA sequencing with Ion PGM System of two *in situ* and two onboard RNA-stabilized samples from different host individuals yielded 23,392,460 reads in total after quality control (Table 2). Transcriptomic assembly using all reads provided 146,985 contigs (*N*_50_, 597 bp; longest contig, 22,476 bp), among which 97,348 contigs were predicted to have 109,253 protein-coding sequences (CDSs). We conducted taxonomic and functional annotations of these CDSs, as well as 49,637 contigs on which no CDSs were predicted. Taxonomic annotations were given to 136,313 (85.8%) sequences (i.e., CDSs and contigs), among which 49,014, 27,321, and 26,141 were assigned to *Thiotrichales*, *Methylococcales*, and *Sulfurovum*, respectively. Functional annotations using the KEGG database annotated 78,748 (49.6%) sequences, which are regarded as genes hereinafter. Among those genes, 64,908 (82.4%) were taxonomically annotated to either of the three major taxa.

**TABLE 2:** Sequencing and initial analysis of rRNA-depleted RNA

Statistic	*In situ*		Onboard	
	mRNA 1	mRNA 2	mRNA 1	mRNA 2
Specimen ID	4	8	2	10
No. of raw reads	6,611,280	4,229,368	6,941,648	7,117,406
No. of quality-controlled reads	5,820,455	4,126,616	6,572,709	6,872,680
Mean read length (bp)	111.2	157.6	140.5	140.9
% Mapped effective reads	74.7	72.1	72.7	74.7
% *Thiotrichales*	18.1	14.2	30.6	28.4
% *Methylococcales*	22.6	28.4	14.6	19.8
% *Sulfurovum*	18.7	16.8	5.3	10.3

**FIG 3:**
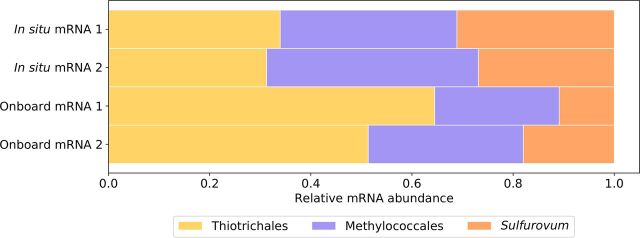
Relative mRNA abundances of the three major taxa. Relative abundances of total mRNA among *Thiotrichales*, *Methylococcales*, and *Sulfurovum* (dominating the *Campylobacterales* episymbionts) are shown. Each bar represents a sample (two *in situ* samples and two onboard samples).

Transcript abundances were quantified by mapping the rRNA-depleted RNA sequences to the assembled contigs and calculating transcripts per million (TPM) values. Total TPM values of the transcripts that were taxonomically annotated to the three major taxa ranged from 58.8% to 69.6% depending on the samples (Table 2). These values were smaller than the rRNA (total RNA) abundances of the same three taxa, likely because of difficulties of taxonomic annotation of mRNA. The relative transcript abundances among the three major taxa are shown in Fig. 3. Compared to the relative rRNA abundances (Fig. 1), the *Sulfurovum* episymbionts showed larger mRNA abundances. This might be because the existence of their closely related genomes in the database enabled more sensitive annotation of *Sulfurovum* mRNA (although *Sulfurovum* had the fewest reference sequences: *Thiotrichales*, 513,398; *Methylococcales*, 268,111; and *Sulfurovum*, 53,394) and/or because the *Sulfurovum* episymbionts produce more mRNAs than the others.

Finally, comparison of the relative abundances between the *in situ* and onboard RNA stabilization methods showed that the two RNA stabilization methods similarly affected the rRNA and mRNA abundances (Fig. 1 and 3), i.e., an overall increase in the *Thiotrichales* profiles but an overall decrease in the *Methylococcales* and *Campylobacterales* (*Sulfurovum*) profiles.

### Metatranscriptomic differences between the *in situ* and onboard RNA stabilization methods.

**FIG 4:**
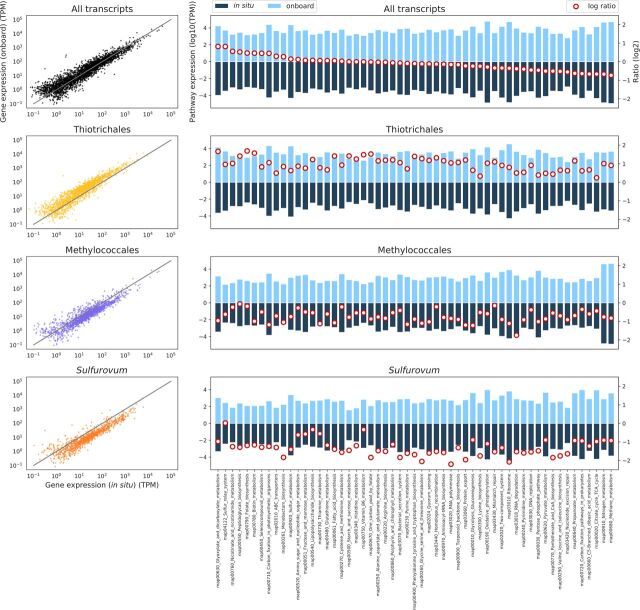
mRNA abundances of genes and pathways compared by the RNA stabilization methods. The left panels show the expression abundance of each gene by the *in situ* (*x* axis) and onboard (*y* axis) RNA stabilization methods in TPM values. From the top to the bottom, data from all transcripts (black) and those assigned to *Thiotrichales* (yellow), *Methylococcales* (purple), and *Sulfurovum* (orange) are shown. Values from the two samples were averaged for each method. The gray line is *x *=* y*. The right panels show the total expression abundance of genes in each KEGG pathway. The upper and lower bars represent results from the onboard and *in situ* RNA stabilization methods, respectively, in log_10_ TPM values. Values from the two samples were averaged for each method. Only pathways with TPM values of >1,000 in at least one sample are shown. The red closed circles represent log ratios of TPM values obtained by both methods.

**FIG 5:**
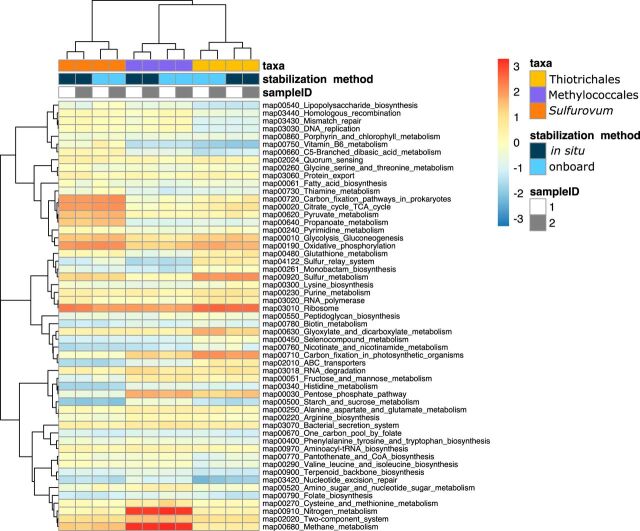
mRNA abundance of pathways normalized for each sample and taxon. The heat map shows the total expression abundance of genes in each KEGG pathway, which was scaled by log_10_ and z-score normalized for each taxon and sample.

We then compared the transcriptomic profiles of the three major taxa, namely, *Thiotrichales*, *Methylococcales*, and *Sulfurovum*, in depth. Gene- and pathway-level relative transcript abundances were examined based on the functional KEGG Orthology (KO) annotations (Fig. 4, the left and right panels show abundances by genes and pathways, respectively). Those of the genes taxonomically assigned to the three major taxa are also shown in the second to fourth rows in the same figure. As described already, when the onboard RNA stabilization method was adopted, the relative abundances of *Thiotrichales* genes increased, and those of *Methylococcales* and *Sulfurovum* genes decreased. What was most notable here was that these shifts seemed to have been caused by transcriptome-wide changes that coincided with rRNA abundance changes but not with large changes in the abundance of specific genes (e.g., those required for metabolism at hydrothermal vents). It may also be notable that some genes and pathways went against the global trends between the two stabilization methods, either by chance or because of the physiological effects of sampling from the deep sea. However, as far as we investigated, we did not see any genes and pathways that exhibited concerted trends against the transcriptome-wide trends in all three major taxa, even among energy and carbon metabolism-related genes and pathways that were likely systematically affected by deep-sea sampling (Fig. 4). To compare mRNA abundance of each sample by cancelling out those transcriptome-wide effects in each taxon, the expression profiles were also visualized by normalizing per each taxon and sample (Fig. 5). First, the expression profiles of the same taxa from the replicate samples were most similar, showing the robustness of the results. Second, the expression profiles of the same taxa were most similar between the *in situ* and onboard RNA-stabilized samples when the transcriptome-wide effects were cancelled out. This also confirmed that the sample retrieval from the deep sea did not substantially affect expression of genes of specific pathways. Thus, we concluded that the transcriptome-wide shift due to deep-sea sampling was the major factor that affected the RNA expression profiles. Specifically, a reasonable explanation would be that the rRNA and mRNA of *Methylococcales* and *Sulfurovum* were globally degraded faster than those of *Thiotrichales* during deep-sea sampling, possibly because of global attenuation of biological activities by depletion of carbon and energy sources and temperature and hydrostatic pressure changes.

**FIG 6:**
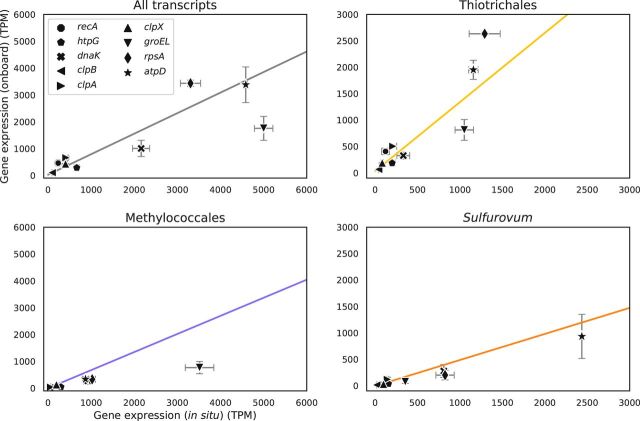
mRNA abundances of stress-responsive genes by RNA stabilization methods. Expression abundance of each stress-responsive gene by the *in situ* (*x* axis) and onboard (*y* axis) RNA stabilization methods is shown. The four panels show data from all transcripts and those assigned to *Thiotrichales*, *Methylococcales*, and *Sulfurovum*. The error bars represent the larger and smaller values of the two samples for each method. The slope is a regression line calculated using the expression abundances of all genes in each major taxon or in total.

We note that previous studies reported gene-specific transcript abundance changes during deep-sea sampling. A study that applied quantitative PCR to analyze the transcript abundance of the *Methylococcales pmoA* gene, which is a key gene in methane oxidation, reported a 3.7-fold decrease in onboard RNA-stabilized samples of deep-sea *S. crosnieri* episymbionts compared to *in situ* RNA-stabilized samples (16). In this study, we observed a 1.82-fold decrease in the TPM values of the *pmoA* gene, and this could be in line with the 1.51-fold transcriptome-wide abundance decrease of *Methylococcales* (Fig. 3 and 4); therefore, the decrease in *pmoA* transcript abundance might actually be a part of the transcriptome-wide effects of deep-sea sampling. It was also reported that the expression of stress response genes (e.g., *recA* and *hsp90*) increases under onboard RNA stabilization (28, 30), although these studies dealt with neither *S. crosnieri* episymbionts nor deep-sea vent microbes. Thus, we analyzed nine genes (*recA*, *dnaK*, *hsp90*, *clpB*, *clpA*, *clpX*, *groEL*, *rpsA*, and *atpD*) for which the expression levels were expected to be increased by environmental stress. In our results, none of the nine genes showed an increase in all three major taxa but collectively followed the transcriptome-wide trends (Fig. 6). A previous study on endosymbionts of Ifremeria nautili, which inhabits deep-sea hydrothermal vents, reported that their 34 h of incubation with few or no electron donors increased the gene expression of the DnaK chaperone system (e.g., *dnaK*) and the Clp/Hsp100 family of ATP-dependent proteins (e.g., *clpA* and *clpX*) (36). However, there are fundamental differences between this study and the previous studies (e.g., 34-h incubation versus 171-min retrieval, endosymbionts versus episymbionts, and taxonomic compositions), and these differences might have led to different transcriptome-wide and gene-specific responses.

### Different metatranscriptomic responses among the three major episymbiotic taxa.

We found that the rRNA and mRNA of *Methylococcales* and *Sulfurovum* were likely rapidly and globally degraded during deep-sea sampling, while those of *Thiotrichales* were kept relatively stable. We were then interested in why *Thiotrichales* episymbionts could keep their rRNA and mRNA more stable than those belonging to the other two major taxa.

We hypothesized that *Thiotrichales* episymbionts utilized intercellular sulfur globules as an energy source to escape the global attenuation of biological activities. Many sulfur-oxidizing bacteria belonging to *Gammaproteobacteria*, including *Thiotrichales*, form intracellular zero-valent sulfur globules as intermediates in sulfur oxidation (35, 37). The TusA and DsrAB proteins are known to be involved in the transfer and oxidization, respectively, of sulfur globules (38–40). In our metatranscriptomic results, we detected the expression of the *tusA* and *dsrAB* genes by *Thiotrichales* episymbionts (TPM values of more than 2,800 and 50, respectively, in all samples). Furthermore, a previous FISH-NanoSIMS analysis revealed that bicarbonate was incorporated into *S. crosnieri* episymbiotic microbial communities without electron donors and that bicarbonate was enriched in short and thin filamentous cells that resembled *Thiotrichales* cells (15). Therefore, the *Thiotrichales* episymbionts were assumed to be able to fix carbon even if electron donors are not supplied using intracellular sulfur globules, and this ability may help them survive even when sulfur compounds are not usable, likely also in a free-living state during horizontal exchange. Notably, the *Sulfurovum* episymbionts, which are also thioautotrophs but showed a decrease in mRNA abundances, expressed neither the *tusA* nor *dsrAB* genes.

### Delineation of the metabolic activities of *S. crosnieri* episymbionts.

Finally, we investigated the physiology and ecology of the *S. crosnieri* episymbiotic community using the *in situ* RNA-stabilized metatranscriptomic data. Functional annotation using the KEGG database revealed the expression of genes of two carbon fixation pathways, genes of a formaldehyde assimilation pathway, and genes that exploit five electron donors (sulfide [HS^−^], thiosulfate [HS_2_O_3_^−^], sulfur [S^0^], methane [CH_4_], and ammonia [NH_4_^+^]) and two electron acceptors (oxygen [O_2_] and nitrate/nitrite [NO_3_^−^/NO_2_^−^]). We could not detect the expression of genes related to hydrogen oxidation or nitrogen fixation; these systems were recently observed in symbiotic chemolithotrophic microbes (41, 42).

**FIG 7:**
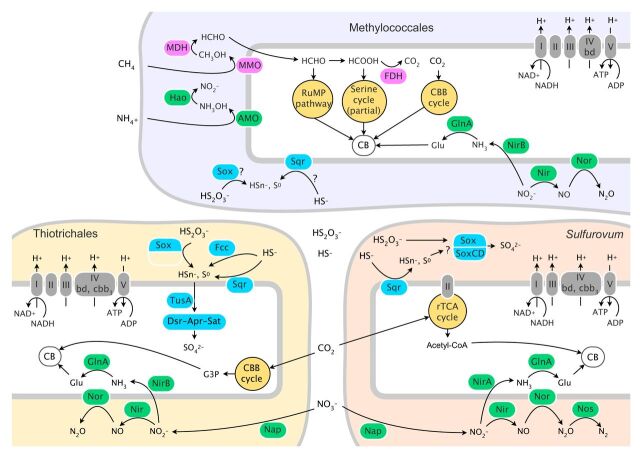
Schematic model of the chemosynthetic metabolisms of the three major *S. crosnieri* episymbiont taxa. Symbols represent proteins involved in each metabolism (blue, sulfur metabolism; pink, methane metabolism; yellow, inorganic carbon fixation; green, nitrogen metabolism). AMO, ammonia monooxygenase; Apr, adenosine phosphosulfate reductase; CB, cellular biosynthesis; CBB cycle, Calvin-Benson-Bassham cycle; Dsr, reverse dissimilatory sulfite reductase; Fcc, flavocytochrome c; FDH, formate dehydrogenase; GlnA, glutamine synthetase; Hao, hydroxylamine; MDH, methanol dehydrogenase; MMO, methane monooxygenase; Nap, two membrane-bound periplasmic nitrate reductases; Nir, nitrite reductase; Nor, a set of membrane-bound nitric oxide reductases; Nos, a set of nitrous oxide reductases; rTCA cycle, reductive tricarboxylic acid cycle; RuMP pathway, ribulose monophosphate pathway; Sat, sulfate adenylyltransferase; Sox, sox enzyme system (Sox and SoxCD, representing the SoxABXYZ complex and the SoxCD complex, respectively); Sqr, sulfide–quinone reductase; TusA, sulfur carrier protein; I, NADH dehydrogenase; II, succinate dehydrogenase; III, complex III cytochrome bc1; IV, bd and cbb3, which are bd-type cytochrome c and cbb3-type cytochrome c, respectively; and V, complex V ATPase.

**(i) Sulfur oxidation.** Substantial expression of genes for oxidation of reduced sulfur compounds by the *Thiotrichales*, *Methylococcales*, and *Sulfurovum* episymbionts was observed (Fig. 7). Those genes included the Sox multiple-enzyme system (*soxABCXYZ*), sulfide:quinone oxidoreductase (*sqr*), flavocytochrome *c* (*fccB*), dissimilatory sulfite reductase (*dsrAB*, functioning in the reverse dissimilatory sulfate reduction pathway), adenylylsulfate reductase (*aprAB*), and sulfate adenylyltransferase (*sat*) genes (see Data Set S1 in the supplemental material). As other sulfur-oxidizing *Gammaproteobacteria* and *Epsilonproteobacteria* do (43, 44), the *Thiotrichales* episymbionts expressed all of these genes except *soxC* (i.e., the Sox system without SoxCD [37, 46]), and the *Sulfurovum* episymbionts expressed *soxABCXYZ* and *sqr*. These observations suggest that both bacteria can oxidize thiosulfate and sulfide to sulfate.

10.1128/mSystems.00551-20.7 DATA SET S1 mRNA abundances of the genes shown in Fig. 7. Download Data Set S1, XLSX file, 0.05 MB. Copyright © 2020 Motoki et al. 2020 Motoki et al. This content is distributed under the terms of the Creative Commons Attribution 4.0 International license.

The *Methylococcales* episymbionts expressed the *soxBXY* and *sqr* genes (TPM values of more than 40 in each of the two *in situ* RNA-stabilized samples), implying that they can also oxidize reduced sulfur compounds. Notably, this possibility of sulfur oxidization by the *Methylococcales* episymbionts of *S. crosnieri* has not been proposed thus far, whereas metagenomic studies of other microbial ecosystems containing methanotrophs (47, 48) and the genome sequences of Methylobacter whittenbury (49) and Methylomarinum vadi (50) indicated that methanotrophs in general can harbor genes for sulfur oxidization.

**(ii) Methane metabolism by *Methylococcales.*** Substantial expression of genes for methane metabolism, specifically for complete methane oxidation and formaldehyde (C_1_ compound) assimilation, by *Methylococcales* episymbionts was observed (Fig. 7 and Data Set S1). These genes included methane monooxygenase (*pmoCAB*), methanol dehydrogenase (*mxaF*), those for dehydrogenation of formaldehyde to formate (*fae*, *mtdAB*, *mch*, and *fhc*), formate dehydrogenase, and hexulose-6-phosphate synthase and hexulose-phosphate isomerase (*hps* and *hpi*) in the ribulose monophosphate (RuMP) pathway. Thus, the *Methylococcales* episymbionts were supposed to be able to oxidize methane to carbon dioxide and assimilate its intermediate, formaldehyde, as a carbon source. On the other hand, only weak expression of six out of nine serine pathway genes (another C_1_ compound assimilation pathway) and the ribulose-1,5-bisphosphate carboxylase/oxygenase (RuBisCO) gene was observed. This implied that the *Methylococcales* episymbionts were mainly composed of type I *Gammaproteobacteria* methanotrophs, which do not possess RuBisCO and serine pathways, consistent with previous phylogenetic analysis of *pmoA* genes (16). Nonetheless, it should be noted that type X methanotrophs, which have RuBisCO and serine pathways (51), might also exist in the community. Expression of the hydroxylamine oxidoreductase gene was also observed, suggesting that *Methylococcales* episymbionts may oxidize ammonia with methane monooxygenase to nitrite via hydroxylamine.

**(iii) Respiration and nitrogen metabolism.** Expression of genes for both oxygen respiration and aerobic denitrification by all three major taxa was observed. Regarding oxygen respiration, the expression of cytochrome *c* oxidase cbb3-type subunit I (*ccoNOPQ*) and cytochrome *c* oxidase bd-type subunit I (*cydAB*) genes was detected in all three major taxa (Data Set S1). Regarding denitrification, the expression of the periplasmic nitrate reductase complex (*napAB*), two cytochrome *cd*-type nitrite reductases (*nirK* or *nirS*), nitric oxide reductase (*nor*), and nitrous oxide reductase (*nosZ*) genes was detected in the *Thiotrichales* and *Sulfurovum* episymbionts, while expression of only the *nirK* and *norBC* genes was detected in *Methylococcales* episymbionts. Thus, *Methylococcales* episymbionts seem to generate nitrous oxide (N_2_O), whereas *Thiotrichales* and *Sulfurovum* episymbionts seem to generate nitrogen (N_2_). The substantial expression of genes involved in denitrification suggests the possibility that the three major taxa utilize nitrate and nitrite as an alternative electron acceptor because of their competition for oxygen.

Expression of cytoplasmic nitrite reductase (*nirBD* or *nirA*) and nitrogen-assimilating glutamine synthetase (*glnA*) genes were detected for all three major taxa. Thus, we assume that nitrite in the periplasm would be transported to and assimilated in the cytoplasm.

**(iv) Carbon fixation.** Expression of the genes of two inorganic carbon fixation pathways among six pathways (43, 52) was observed in the *Thiotrichales* and *Sulfurovum* episymbionts. The *Thiotrichales* episymbionts expressed genes encoding the Calvin-Benson-Bassham (CBB) cycle, including the *RuBisCO* gene. The *Sulfurovum* episymbionts expressed genes encoding the reductive tricarboxylic acid (rTCA) cycle, including *oor*, *frd*, and *acl* genes. Thus, the CBB and rTCA cycles are likely utilized by the *Thiotrichales* and *Sulfurovum* episymbionts, respectively, which is consistent with the physiological characteristics of the cultured strains that belong to *Gammaproteobacteria* and *Epsilonproteobacteria*, respectively (43, 53–55).

Other notable observations would include that *Thiotrichales* showed relatively high expression of glyoxylate and dicarboxylate metabolism pathway genes and *Methylococcales* showed relatively high expression of the pentose phosphate pathway genes (Fig. 5).

### Conclusion.

In this study, we performed direct metatranscriptome analysis of episymbionts of *S. crosnieri* by applying the *in situ* RNA stabilization equipment. To our knowledge, this is the first study that applied and systematically compared the different RNA stabilization approaches to deep-sea symbiotic microbial communities.

Comparison between the *in situ* and onboard RNA stabilization methods is highly demanded in applying the metatranscriptomic approach to deep-sea microbial communities, which can hardly be directly investigated by laboratory experiments, for three reasons. First, the onboard RNA stabilization method can give biased results that are different from those obtained by *in situ* RNA stabilization. Second, by doing so, the transcriptome-wide and gene-specific effects of deep-sea sampling can be discriminated. Third, by positively regarding such methodological differences as environmental perturbation, comparative metatranscriptomics may provide new insights into the physiology and ecology of microbial community members, as we did in this study. Last but not least, we note that these three factors would also be considered in metatranscriptomic analyses of other access-limited ecosystems.

The direct metatranscriptome analysis showed that methane utilization and sulfur oxidation are the main energy metabolisms of the *S. crosnieri* episymbionts. The dominant yet uncultured *Thiotrichales*, *Methylococcales*, and *Sulfurovum* expressed genes that encode almost all the components of the pathways for these chemoautotrophic systems. It may be notable that no expression of hydrogenase genes was detected in this study, while the genome of *Sulfurovum* sp. strain NBC37-1, which was isolated from an *in situ* sampler deployed on the same NBC hydrothermal vent site (i.e., not from the *S. crosnieri* body surface), carries four hydrogenase genes (55, 56). Whether the *Sulfurovum* episymbionts contribute to hydrogen flux will require further studies.

The results of the taxonomic clustering analysis indicated that the composition of the episymbiotic microbial populations is more complex than previously thought, where each of the three taxonomic clades likely contained diverse species. We anticipate that comparative analysis of metagenome-assembled genomes of the *S. crosnieri* episymbionts powered by long-read single-molecule sequencing technologies will help better understand their true biodiversity, immigration, and evolution within and among different hydrothermal vent habitats in the Okinawa Trough.

## MATERIALS AND METHODS

### Sample collection and RNA stabilization.

*S. crosnieri* individuals were collected from a single colony at the NBC hydrothermal vent site in the Iheya North field in the Okinawa Trough (27° 47.45′ N, 126° 53.80′ E, 982-m depth; see Fig. S1 in the supplemental material) during dive 1773 of the remotely operated vehicle (ROV) *HyperDolphine* on 18 January 2015. The details of the ROV are shown and described in Fig. S2. The seawater temperature of *S. crosnieri* habitats around the site was 5.7°C on average. All procedures were performed following the manufacturer’s instructions and with default settings unless otherwise noted.

10.1128/mSystems.00551-20.2 FIG S2 Mounted equipment on the ROV *HyperDolphine*. The photograph shown in the left was taken by Masayuki Miyazaki. Download FIG S2, EPS file, 1.2 MB. Copyright © 2020 Motoki et al. 2020 Motoki et al. This content is distributed under the terms of the Creative Commons Attribution 4.0 International license.

10.1128/mSystems.00551-20.3 FIG S3 Ecological indices of the *S. crosnieri* episymbiotic microbial communities of the *in situ* and onboard RNA-stabilized samples. (a) Rarefaction curves. (b to d) Box-whisker plots of OTU richness (b), Faith’s phylogenetic diversity (c), and Shannon diversity (d). Points represent individual samples. The lines in the boxes represent medians. The boxes and whiskers represent ranges and interquartile ranges, respectively. Mann-Whitney *U* tests were performed (n.s., not significant; *, *P* < 0.05). Download FIG S3, EPS file, 0.1 MB. Copyright © 2020 Motoki et al. 2020 Motoki et al. This content is distributed under the terms of the Creative Commons Attribution 4.0 International license.

10.1128/mSystems.00551-20.4 FIG S4 PCoA based on weighted UniFrac among the *in situ* and onboard RNA-stabilized samples. Variance explained by each PC is indicated on the axis. Plots represent results from individual samples of the *in situ* (dark blue) and onboard (light blue) RNA stabilization methods. Download FIG S4, EPS file, 0.04 MB. Copyright © 2020 Motoki et al. 2020 Motoki et al. This content is distributed under the terms of the Creative Commons Attribution 4.0 International license.

*In situ* RNA stabilization was conducted by following the procedure described by Watsuji et al. (16). Briefly, *S. crosnieri* individuals were collected in a sample container of the ROV using a suction sampler at the sampling site (Fig. S2). Then, the seawater in the container was replaced with aqueous sulfate salt solution (25 mM sodium citrate, 10 mM EDTA, and 700 g/liter ammonium sulfate; pH 5.2; colored with phenol red for visibility; prepared at 4°C before the ROV dive) by spontaneous diffusion in 9 min (sampling time, 11:46, stabilization time, 11:55 [Japan standard time {JST}]). The aqueous sulfate salt solution, which was proved to inhibit RNase activity (57), was used to provide a large amount of RNA stabilization solution. The *S. crosnieri* samples were then retrieved by a research vessel. The samples were washed three times using chilled aqueous sulfate salt solution to remove possibly contaminating bacteria that did not firmly attach to the *S. crosnieri* bodies. The setae covered with episymbionts were dissected from the host individuals and stored in RNAlater (Qiagen) at 4°C overnight and −80°C subsequently.

For onboard RNA stabilization, *S. crosnieri* individuals were collected in another sample container using another suction sampler during the same dive. To remove air and to keep the animals alive until retrieval by being kept in cold seawater, the container was prefilled with chilled filtered seawater and kept on −20°C ice packs before the dive. After collection, the *S. crosnieri* individuals were retrieved by a research vessel. The samples were washed three times using chilled sterile artificial seawater (25 g/liter NaCl, 4.2 g/liter MgCl_2_·6H_2_O, 3.4 g/liter MgSO_4_·7H_2_O, 0.5 g/liter KCl, 0.7 g/liter CaCl_2_·2H_2_O, and 14 mg/liter K_2_HPO_4_; pH 6.8) and submerged in aqueous sulfate salt solution. The duration from sample collection to submergence was 171 min (sampling time, 11:39; stabilization time, 14:30 [JST]). The setae covered with episymbionts were dissected from the host individuals and stored in RNAlater at 4°C overnight and −80°C subsequently. For this study, seta samples from seven *S. crosnieri* individuals each from the *in situ* and onboard RNA stabilization methods (i.e., from 14 individuals in total) were used. The carapace lengths and widths of the 14 individuals ranged from 30 to 48 mm and from 25 to 37 mm, respectively.

### RNA extraction.

Total RNA extraction (for 16S rRNA analysis) and subsequent rRNA-depleted RNA preparation (for metatranscriptomic analysis) were conducted. Total RNA extraction was conducted using all 14 seta samples. RNA was extracted using an RNA PowerSoil Total RNA isolation kit (Mo Bio), treated using DNase I (Qiagen) for 10 min at room temperature, and recovered using an RNeasy minikit (Qiagen). The quantity and quality of the RNA were measured using a Qubit RNA HS assay kit with a Qubit 1.0 fluorometer (Invitrogen) and an Agilent RNA 6000 Pico kit with an Agilent 2100 Bioanalyzer (Agilent). We did not observe eukaryotic 18S and 28S rRNA peaks due to host RNA contamination using the Bioanalyzer. rRNA was depleted from four of the total RNA samples that had good quantities and qualities (two each from the *in situ* and onboard RNA stabilization methods). The samples were treated with the Ribo-Zero rRNA Removal kit for Gram-Negative Bacteria (Epicentre) and purified using RNA Clean & Concentrator (Zymo Research). Using a Bioanalyzer and Qubit, rRNA depletion was confirmed, and the RNA quantity was measured.

The RNA samples were kept at −80°C until sequencing.

### Sequencing and initial quality check.

Sequencing of total RNA was conducted using 13 samples that comprised six *in situ* and seven onboard RNA-stabilized samples (one *in situ* sample was not used because of a quality problem). The RNA quantities and RNA integrity number (RIN) values ranged from 301.8 to 500.8 ng and from 5.6 to 6.7, respectively. Barcoded cDNA libraries were obtained using the Ion Total RNA-Seq kit v2 and Ion Xpress RNA-Seq Barcode 1–16 kit (Life Technologies). The quantities and library sizes of the cDNA libraries were measured using a Quant-iT dsDNA HS assay kit with a Qubit 2.0 fluorometer and an Agilent High Sensitivity DNA kit with an Agilent 2100 Bioanalyzer, respectively. Each cDNA library was diluted to 50 pM. Then, 5 μl of each solution was pooled to prepare two solutions so that each of the pooled solutions contained either the six *in situ* or seven onboard RNA-stabilized samples. From each of the two pooled solutions, 25 μl was loaded onto an Ion 318 v2BC Chip using an Ion PGM Hi-Q Chef kit with an Ion Chef system. Two sequencing runs were conducted using the Ion PGM Hi-Q Sequencing kit for 200-bp libraries with the Ion PGM System (Life Technologies). Sequencer-specific errors were corrected with Karect (58) (*-celltype=haploid -matchtype=edit*). Low-quality sequence regions and short reads were trimmed (*-trim_qual_left 20 -trim_qual_right 20*) and removed (*-min_len 200*), respectively, using PRINSEQ v0.20.4 (59).

Sequencing of the four rRNA-depleted RNA samples was conducted as follows. The RNA quantities and RIN values ranged from 316.8 to 436.8 ng and from 5.9 to 6.9, respectively. cDNA libraries were obtained using the Ion Total RNA-Seq kit v2. Quantities and library sizes were measured as described above. Each cDNA library was diluted to 20 pM. From each of the four solutions, 25 μl was loaded onto an Ion 318 v2BC Chip using an Ion PGM Hi-Q View Chef kit with an Ion Chef system. Four sequencing runs were conducted using the Ion PGM Hi-Q View Sequencing kit for 200-bp libraries with the Ion PGM System. Sequencer-specific errors were corrected with Karect (*-celltype=haploid -matchtype=edit*). Low-quality sequence regions and short reads were trimmed (*-trim_qual_left 26 -trim_qual_right 26*) and removed (*-min_len 50*), respectively, using PRINSEQ. Note that the low-quality filter was set at a stricter level here for subsequent metatranscriptome assembly.

### rRNA analysis.

Reads generated by the two total RNA sequencing runs (i.e., those from 13 samples) were used for 16S rRNA analysis. Taxonomic annotation of each read was performed using BLASTn from BLAST+ 2.7.1 (60) against SILVA SSURef NR99 123 (61) (http://www.arb-silva.de/) and by retrieving the top hit that satisfied the criteria E value ≤ 1E−20, alignment length ≥ 180 bp, and identity ≥ 97%. If the top hit was attributed to multiple SILVA identifiers typically because a conserved region was sequenced, the most frequent identifier from the same stabilization method was chosen based on Occam’s razor. The relative abundance of each SILVA identifier was quantified by the assigned read numbers. Then, the SILVA sequences were clustered with a 95% identity threshold using UCLUST v1.2.22q (62, 63). Each cluster was designated an operational taxonomic unit (OTU). The abundance of each OTU was calculated as the sum of those of the contained SILVA identifiers. Alpha diversity (OTU richness, Shannon diversity, and Faith’s phylogenetic diversity) and beta diversity (weighted UniFrac) were calculated using skbio package v0.5.6 (scikit-bio.org). To calculate Faith’s phylogenetic diversity and weighted UniFrac, representative sequences of OTUs selected by UCLUST were aligned using MAFFT v7.470 (64) and phylogenetic trees were reconstructed using FastTree v2.1.11 (65) with the GTR+CAT model. Principal coordinate analysis (PCoA) was conducted based on weighted UniFrac using the prcomp package in R.

### Metatranscriptomic analysis.

Reads from rRNA-depleted RNA sequencing were used for metatranscriptomic analysis. Because reference genomes of the episymbionts were not available, all reads generated by the four sequencing runs were merged and fed into the transcript assembly using Trinity v2.3.2 (45) (*–min_contig_length 300*). For quantification, we used RSEM v 1.2.30 (66) and Bowtie2 v 2.2.9 (67) through the align_and_estimate_abundance.pl script in Trinity (*–est_method RSEM –aln_method bowtie2*) to map the rRNA-depleted RNA sequencing reads to the assembled transcript contigs and quantify their abundances as transcripts per million (TPM) values.

Protein-coding sequences (CDSs) on each transcript contig were predicted by TransDecoder (http://transdecoder.github.io/). The taxonomic and functional annotations of the CDSs were conducted using BLASTp (E-value cutoff of 1E−5) against the NCBI nonredundant (NCBInr) and Kyoto Encyclopedia of Genes and Genomes (KEGG) (68) (as of 12 March 2018) databases, respectively. Because the transcript contigs could contain partial genes that were missed by the CDS prediction, contigs without predicted CDSs were also queried against the NCBInr and KEGG databases for taxonomic and functional annotations, respectively, using BLASTx (E-value cutoff of 1E−5). For the taxonomic annotation using NCBInr, NCBI taxonomic identifiers (TaxIDs) were assigned to the BLAST hits by cross-referencing against the NCBI database (as of 26 March 2019; ftp://ftp.ncbi.nlm.nih.gov/pub/taxonomy/accession2taxid). Then, the top hit in terms of E value (with bit scores in a tie) among the hits assigned to 1,751 TaxIDs that belonged to the order *Thiotrichales* or *Methylococcales* or genus *Sulfurovum* was retrieved (because >99% rRNA sequences assigned to the order *Campylobacterales* were assigned to *Sulfurovum* at the genus level [Fig. S1] and *Sulfurovum* did not belong to *Campylobacterales* but to unclassified *Epsilonproteobacteria* in NCBInr [as of 7 March 2018], the genus *Sulfurovum* was used instead of *Campylobacterales* for mRNA taxonomic assignment). For the functional annotation using KEGG, the KEGG Orthology (KO) number of the top hit was used if one was assigned.

### KEGG orthology, module, and pathway analyses.

For each sample, a TPM value of each KO group was calculated as the sum of TPM values of CDSs and contigs that were functionally annotated to that KO. Likewise, the TPM value of each KEGG module and KEGG pathway was calculated as the sum of TPM values of KOs that belonged to each.

Analyses of the transcript abundances of the following genes were performed using the following KOs: *pmoA* by K10944, *recA* by K03553, *rpsA* by K02945, *atpD* by K02112, *hsp90* by K04079, *clpB* by K03695, *clpA* by K03694, *clpX* by K03544, *dnaK* by K04043, *groEL* by K04077, and *hupL* by K06281. Analyses of the transcript abundances of the following functional categories were performed using the following KEGG modules (M numbers) and KOs: sulfur metabolism by M00176, M00596, M00595, K17229, K17230, and K17218; methane metabolism by M00174, M00346, M00345, M00344, M00140, K00122, K10713, K10714, and K01499; carbon fixation metabolism by M00165, M00173, M00579, M0376, M0375, M0374, M0377, and M0620; oxygen respiration by M00153, M00417, M00416, M00156, M00157, and M00159; nitrogen metabolism by M00175, M00531, M00530, M00529, and M00804; and adhesion mechanism by M00330 and K12549.

### Ethics approval and consent to participate.

The locations for sample collection were not privately owned or protected in any way, and no specific permits were required for the described field studies and sample collection. The field studies did not involve any endangered or protected species.

### Data availability.

The sequence data and assembled contigs were deposited in the DDBJ/ENA/GenBank database under BioProject identifier (ID) PRJDB9200. The bioinformatic scripts are available in github (https://github.com/kaorimo/MetatraAnalysis_mSystems2020).
