# Supplementary material for: Metatranscriptomics by In Situ RNA Stabilization Directly and Comprehensively Revealed Episymbiotic Microbial Communities of Deep-Sea Squat Lobsters
Source: mSystems. 2020 Oct 6;5(5):e00551-20. doi: 10.1128/mSystems.00551-20 (PMC8534475; doi:10.1128/mSystems.00551-20)
Supplement: TABLE S1 [file msystems.00551-20-st001.docx]

|  | ***Arcobacter*** | ***Campylobacter*** | ***Sulfurimonas*** | ***Sulfurospirillum*** | ***Sulfurovum*** |
| --- | --- | --- | --- | --- | --- |
| ***In situ* total RNA 1** | 0.000 (0) | 0.000 (0) | 0.000 (1) | 0.000 (1) | 0.093 (719) |
| ***In situ* total RNA 2** | 0.000 (6) | 0.000 (0) | 0.000 (2) | 0.000 (0) | 0.138 (3803) |
| ***In situ* total RNA 3** | 0.000 (4) | 0.000 (0) | 0.000 (3) | 0.000 (0) | 0.193 (4911) |
| ***In situ* total RNA 4** | 0.000 (7) | 0.000 (0) | 0.000 (1) | 0.000 (8) | 0.135 (5151) |
| ***In situ* total RNA 5** | 0.000 (1) | 0.000 (0) | 0.000 (2) | 0.000 (1) | 0.140 (3772) |
| ***In situ* total RNA 6** | 0.000 (1) | 0.000 (0) | 0.000 (2) | 0.000 (4) | 0.181 (4600) |
| **Onboard total RNA 1** | 0.000 (0) | 0.000 (0) | 0.000 (1) | 0.000 (2) | 0.110 (1808) |
| **Onboard total RNA 2** | 0.000 (0) | 0.000 (0) | 0.000 (4) | 0.000 (4) | 0.109 (1662) |
| **Onboard total RNA 3** | 0.000 (5) | 0.000 (0) | 0.000 (4) | 0.000 (1) | 0.095 (3646) |
| **Onboard total RNA 4** | 0.000 (9) | 0.000 (1) | 0.000 (3) | 0.000 (4) | 0.108 (2559) |
| **Onboard total RNA 5** | 0.000 (9) | 0.000 (2) | 0.000 (1) | 0.000 (1) | 0.095 (5575) |
| **Onboard total RNA 6** | 0.000 (3) | 0.000 (0) | 0.000 (1) | 0.000 (0) | 0.102 (1635) |
| **Onboard total RNA 7** | 0.000 (3) | 0.000 (0) | 0.000 (3) | 0.000 (2) | 0.063 (1071) |
