# Supplementary material for: Metatranscriptomics by In Situ RNA Stabilization Directly and Comprehensively Revealed Episymbiotic Microbial Communities of Deep-Sea Squat Lobsters
Source: mSystems. 2020 Oct 6;5(5):e00551-20. doi: 10.1128/mSystems.00551-20 (PMC8534475; doi:10.1128/mSystems.00551-20)
Supplement: TABLE S2 [file msystems.00551-20-st002.docx]

| **OTU No.** | **Taxonomy** | **# SILVA identifier** |
| --- | --- | --- |
| 13 | Proteobacteria; Gammaproteobacteria; Thiotrichales; Thiotrichaceae; uncultured | 18 |
| 13 | Proteobacteria; Gammaproteobacteria; Thiotrichales; Thiotrichaceae; *Cocleimonas* | 16 |
| 13 | Proteobacteria; Gammaproteobacteria; Thiotrichales; Thiotrichaceae; None | 1 |
| 17 | Proteobacteria; Gammaproteobacteria; Methylococcales; Marine Methylotrophic Group 2; None | 24 |
| 55 | Proteobacteria; Gammaproteobacteria; Thiotrichales; Thiotrichaceae; uncultured | 8 |
| 123 | Proteobacteria; Epsilonproteobacteria; Campylobacterales; Helicobacteraceae; *Sulfurovum* | 30 |
| 134 | Proteobacteria; Epsilonproteobacteria; Campylobacterales; Helicobacteraceae; *Sulfurovum* | 42 |
| 180 | Proteobacteria; Gammaproteobacteria; Thiotrichales; Thiotrichaceae; *Thiothrix* | 12 |
| 217 | Proteobacteria; Epsilonproteobacteria; Campylobacterales; Helicobacteraceae; Sulfurovum | 14 |
| 250 | Proteobacteria; Gammaproteobacteria; Thiotrichales; Thiotrichaceae; uncultured | 11 |
| 250 | Proteobacteria; Gammaproteobacteria; Thiotrichales; Thiotrichaceae; *Cocleimonas* | 1 |
| 253 | Proteobacteria; Gammaproteobacteria; Thiotrichales; Thiotrichaceae; uncultured | 4 |
| 254 | Proteobacteria; Gammaproteobacteria; Thiotrichales; Thiotrichaceae; uncultured | 3 |
| 268 | Proteobacteria; Gammaproteobacteria; Methylococcales; Marine Methylotrophic Group 2; None | 2 |
| 286 | Proteobacteria; Gammaproteobacteria; Methylococcales; pLW-20; None | 2 |
| 294 | Proteobacteria; Epsilonproteobacteria; Campylobacterales; Helicobacteraceae; *Sulfurovum* | 22 |
| 306 | Proteobacteria; Gammaproteobacteria; Thiotrichales; Piscirickettsiaceae; *Cycloclasticus* | 1 |
| 306 | Proteobacteria; Epsilonproteobacteria; Campylobacterales; Helicobacteraceae; *Sulfurovum* | 1 |
| 340 | Proteobacteria; Gammaproteobacteria; Methylococcales; Marine Methylotrophic Group 2; None | 3 |
| 352 | Proteobacteria; Gammaproteobacteria; Thiotrichales; Thiotrichaceae; *Thiothrix* | 1 |
| 382 | Proteobacteria; Epsilonproteobacteria; Campylobacterales; Helicobacteraceae; *Sulfurovum* | 1 |
